# Efficacy of N-Acetyl Cysteine in Traumatic Brain Injury

**DOI:** 10.1371/journal.pone.0090617

**Published:** 2014-04-16

**Authors:** Katharine Eakin, Renana Baratz-Goldstein, Chiam G. Pick, Ofra Zindel, Carey D. Balaban, Michael E. Hoffer, Megan Lockwood, Jonathan Miller, Barry J. Hoffer

**Affiliations:** 1 Department of Neurosurgery, Case Western Reserve University School of Medicine, Cleveland, Ohio, United States of America; 2 Department of Anatomy and Anthropology, Sackler School of Medicine, Tel-Aviv University, Tel-Aviv, Israel; 3 Department of Otolaryngology, Neurobiology, Communication Sciences and Disorders, and Bioengineering, University of Pittsburgh, Pennsylvania, United States of America; 4 Department of Otolaryngology, Spatial Orientation Center, Naval Medical Center San Diego, San Diego, California, United States of America; 5 Graduate Program in Neuroregeneration, Taipei Medical University, Taipei City, Taiwan; University of South Florida, United States of America

## Abstract

In this study, using two different injury models in two different species, we found that early post-injury treatment with N-Acetyl Cysteine (NAC) reversed the behavioral deficits associated with the TBI. These data suggest generalization of a protocol similar to our recent clinical trial with NAC in blast-induced mTBI in a battlefield setting [1], to mild concussion from blunt trauma. This study used both weight drop in mice and fluid percussion injury in rats. These were chosen to simulate either mild or moderate traumatic brain injury (TBI). For mice, we used novel object recognition and the Y maze. For rats, we used the Morris water maze. NAC was administered beginning 30–60 minutes after injury. Behavioral deficits due to injury in both species were significantly reversed by NAC treatment. We thus conclude NAC produces significant behavioral recovery after injury. Future preclinical studies are needed to define the mechanism of action, perhaps leading to more effective therapies in man.

## Introduction

Traumatic brain injury (TBI) is a major public health issue that affects 1.7 million Americans each year [2] and has been termed a silent epidemic by the CDC. Many survivors experience prolonged or even permanent neurocognitive dysfunction, with lasting changes in cognition, motor function, and personality [3]. A conservative estimate is that 3.2 million Americans, or 1.5% of the population, currently live with long-term disabilities after TBI, and these disabilities are estimated to cost $9.2 billion in lifetime medical costs and $51.2 billion in productivity losses [4].

The pathophysiology of TBI is divided into primary and secondary injury processes. Primary injury refers to the direct physical trauma to the brain from impact force or penetrating injury. Secondary injury involves a cascade of molecular mechanisms that are initiated at the time of trauma and evolves in the hours and days after the traumatic event. These mechanisms include glutamatergic excitotoxicity, free-radical injury to cell membranes, electrolyte imbalances, mitochondrial dysfunction, inflammatory responses, apoptosis, and secondary ischemia from vasospasm [5], [6], [7], [8]. Since these processes are believed to be partially responsible for the progressive neurological impairment after TBI, the development of effective therapeutic strategies capable of arresting secondary injury-induced damage has become a focus of intense research activity over the last two decades, both in clinical and preclinical settings.

*N*-Acetyl-L-cysteine (NAC) is the active agent in Mucomyst, a US Food and Drug Administration approved medication with a forty-year safety history. There is also literature on NAC as a neuroprotective agent in preclinical models of central and peripheral nervous injury. NAC has been shown to have antioxidant and neurovascular-protective effects after TBI [9], [10], [11]. When combined with minocycline, NAC treatment following controlled cortical impact (CCI) increased levels of anti-inflammatory M2 microglia in white matter tracts [12]. Such studies however, have been primarily at the biochemical and cellular levels, rather than focusing on behavioral parameters.

We recently conducted, in an active theatre of war, a study demonstrating that NAC, in addition to standard symptomatic therapy, has beneficial effects on the severity and resolution of auditory, vestibular and cognitive function sequelae after blast induced mild TBI (mTBI) in military personnel [1]. In this paper, we sought to determine the efficacy of NAC in two different rodent models of TBI to determine the generality of any effects and allow for future mechanistic studies. The studies were organized first to examine the efficacy of NAC in blunt trauma, to study the potential for transitioning NAC. In order to further study this potential, in the second group of animals, NAC was combined with topiramate. The rationale for this is the fact that in any future human clinical trial with NAC a standard of care for headache could not be withheld irrespective of how the experimental protocol is organized. Behavioral parameters of learning and memory were used as end points to correlate with the clinical trial of NAC in theater cited above. We used a weight drop (WD) protocol in mice and a midline fluid percussion injury (FPI) protocol in rats. The WD protocol models “mild” TBI (mTBI) where there is little evidence for gross anatomical disruption of brain tissue whereas the FPI protocol simulates “moderate” TBI. In the article by Hoffer [1] it was reported that NAC treatment was supplemented, in some patients, with standard treatment with topiramate, a non-narcotic medication used to treat post-traumatic headache symptomatically. Hence, we added treatment with topiramate concurrently with NAC in the mouse study of closed head concussion.

## Materials and Methods

### Experiment 1: Fluid Percussion Injury in rats

#### Animals

Male Sprague-Dawley rats (Harlan Laboratories Inc., Indianapolis, IN) weighing between 350 and 400 grams were used. Animals were housed under a 12 hour light/dark cycle and provided with food and water *ad libitum*. All animal procedures were conducted in accordance with guidelines reviewed and approved by the Institutional Animal Care and Use Committee of Case Western Reserve University, and in accordance with the Guide for the Care and Use of Laboratory Animals as adopted and promulgated by the U.S. National Institutes of Health.

#### Fluid Percussion Injury

The fluid-percussion injury (FPI) device used to produce experimental TBI was identical to that described in detail by others [13]. All rats were surgically prepared for midline FPI. The animals were randomly assigned to one of three treatment groups: Sham (*n = *9), TBI (*n* = 9), or TBI-NAC (*n* = 8). Under 2% isoflurane anesthesia, a 4.8 mm diameter burr hole was performed midline between the coronal and lambdoid sutures, and a Luer-Loc hub was affixed to the perimeter of the burr hole using cyanoacrylate. Dental acrylic and two small nickel-plated screws were used to anchor a hub to the skull. Twenty-four hours later, at the time of injury, the rats were anesthetized, the surgical site was exposed, and the animals were connected to the injury device. The force of the injury administered was between 1.82–1.95 atmospheres of pressure (atm) which correlates to a moderate degree of injury. Sham animals were connected to the injury device but no injury was delivered. NSAIDs were used for postoperative analgesia.

#### Materials and Drug Treatment

NAC (Sigma Aldrich, St. Louis MO) was dissolved in 0.9% sterile saline and the pH was adjusted to 7.2 using hydrochloric acid. Animals assigned to TBI-NAC received the drug at 30 minutes post-injury, injected at a dose of 50 mg/kg intraperitoneally (ip), and then once every 24 hours over the next three days, for a total of four doses based on previous reports [14], [15]. Animals in the Sham and TBI groups received i.p. injections with 0.9% sterile saline using the same dosing paradigm.

#### Cognitive Assessment

*Morris water maze - Hidden Platform*. Spatial learning and memory was assessed using the Morris water maze (MWM) task on post injury days (PID) 10–14. The protocol for hidden platform testing has been described in detail elsewhere [16]. Briefly, a circular tank 180 cm in diameter, 45 cm in height, was filled with water maintained between 25–28°C. Maze performance and video tracking for each animal was measured using EthoVision XT 8.5 (Noldus Information Technology Inc., Leesburg, VA). The goal of the task is to locate a hidden submerged platform that is 15 cm in diameter located 2 cm below the surface of the water. The location of the platform was fixed across all the trials during hidden platform testing. Distinct visual cues on the walls of the maze room, which remained constant across all trials, provided spatial references to assist the animals in locating the hidden platform. Rats were tested for four trials per day over four days on PID 10–13. In each trial, the rat was placed into the water from one of four cardinal directions in a pseudorandom order and allowed up to 120 seconds to locate the platform. If the animal did not locate the platform within 120 seconds, they were gently guided to it by the experimenter. All animals were allowed to remain on the platform for 30 seconds before being removed from the tank and placed in a heated incubator for 10 minutes until the next trial.

*Morris water maze – Probe Trial*. A probe trial was performed 24 hours after the last day of hidden platform testing, on PID 14. Each animal was allowed to swim for 30 seconds in the pool with the platform removed to determine the number of times the animal crossed the platform zone, defined as 2× the diameter of the platform (i.e., 30 cm diameter, or an additional 7.5 cm radius beyond the platform perimeter).

*Morris water maze- Visible Platform*. A visible platform test was performed immediately following the probe trial, on PID 14. The water level was lowered in the tank so that the platform surface was 1.5 cm above water level. The animal was allowed to swim for 60 seconds in the pool to locate the platform. After the platform was located, the animal was allowed to remain on the platform for 30 seconds. If the animal did not locate the platform within 60 seconds, it was gently guided to it by the experimenter and then allowed to remain for 30 seconds. A second trial was immediately conducted, and the latency to reach the platform was measured as a test for visual acuity and swim strength.

#### Statistical Analysis

Statistical analyses were performed using IBM, SPSS Statistics 20. Student's t-test was used to compare the duration of the suppression of the righting reflex between the two injured groups. One-way or repeated-measures ANOVA were used to determine overall groups differences during MWM testing. Fisher's LSD post hoc test was used where appropriate. A p-value of 0.05 or less was defined as statistically significant.

### Experiment 2: Weight Drop in Mice

#### Animals

Male ICR mice (6–8 weeks age and 30–40 g weight) were purchased from Harlan Sprague-Dawley (HSD Jerusalem), Israel, and thereafter bred and raised within the vivarium. Animals were housed 3–5 per cage with *ad libitum* access to food and water on a 12 hour light/dark cycle at 22±1°C. All experimental manipulations were undertaken during the light phase of the cycle. Experimental procedures and housing conditions were approved by the Institutional Animal Care and Use Committee of Tel Aviv University (M-10-030), and in accordance with the Guide for the Care and Use of Laboratory Animals as adopted and promulgated by the U.S. National Institutes of Health. A minimum number of animals were used and all efforts were made to minimize potential suffering. Each animal was used for only one experiment.

#### Weight Drop Injury

On the day of injury, mice were randomly assigned to one of 4 treatment groups, Sham-Vehicle (*n* = 9), Sham-Drug (NAC + topiramate; *n* = 6), TBI-Vehicle (*n* = 9), or TBI-Drug (NAC + topiramate; *n* = 8) groups. Animals were anesthetized lightly with isoflurane and then placed under the weight drop device. This device consists of a cylindrical 30 g weight with a rounded tip which is dropped through a vertical metal guide tube (13 mm in diameter and 80 cm long). Each mouse was placed in a right lateral decubitus position on a molded foam pad with the right temporal region, between the corner of the eye and the ear, directly under the guide tube. TBI was produced by releasing the weight from the top of the tube onto the scalp. The foam pad used to support the head of the mouse allowed some anterior/posterior motion in the absence of any rotational head movement [17], [18]. Sham-injured mice underwent identical treatment but no weight was dropped.

#### Materials and Drug Treatment

NAC (Sigma Aldrich Israel Ltd., Rehovot, Israel) was prepared as a suspension in sterile 0.9% saline at a concentration of 100 mg/10 ml and pH adjusted to 7.2. Topiramate (Sigma Aldrich Israel Ltd., Rehovot, Israel) was prepared as a suspension in 2% dimethyl sulfoxide (DMSO; vehicle), to provide final concentration of 30 mg/10 ml. Both drugs were administered intraperitoneally (i.p.) in a volume of 0.1 ml/10 g body weight. Thus, the dose after NAC was 100 mg/kg and for topiramate was 30 mg/kg. These doses were chosen to parallel the doses used in the clinical trial in human blast trauma [1]. One hour after injury or sham-injury, animals were treated with single doses of either Vehicle (DMSO) or Drug (NAC + topiramate).

#### Cognitive Assessment

At 7 and 30 days after WD or sham-injury, animals were assessed using two behavioral tests: novel object recognition (NOR) and the Y maze.

*Novel Object Recognition.* An object recognition task was used to assess recognition memory [19]. Mice were individually habituated to an open field box (59×59×20 cm) for 5 minutes, 24 hours before the test. During the acquisition phase, two identical objects (A and B), which were sufficiently heavy and high to ensure that mice could neither move nor climb over them, were placed in a symmetric position within the chamber. Each animal was then placed in the box and allowed to explore the objects for 5 minutes. Twenty-four hours after the acquisition phase, one object (A or B randomly) was substituted for a novel one (C) and exploratory behavior was again evaluated for 5 minutes. The open field box and all objects were thoroughly cleansed using 70% ethanol between sessions to preclude odor recognition. Exploration of an object was characterized as rearing on it or sniffing it at a distance of less than 2 cm and/or touching it with the nose. Successful recognition was revealed by preferential exploration of the novel object [20]. Discrimination of visual novelty was assessed by a preference index [21], determined as: (time near the new - time near the old object)/(time near the new + time near the old object).

*Y maze paradigm.* The Y maze test was used to assess spatial memory [20]. This task takes advantage of the preference of rodents to explore novel rather than familiar places. The Y maze was constructed of black Perspex and comprised of three arms (8×30×15 cm at an angle of 120° from the others), each distinguished by the presence of a different visual cue (triangle, square, or circle). One arm was randomly selected as the ‘start’ arm and this remained constant for each animal on both trials. During the initial trial, of 5 minutes duration, one of the two remaining arms was randomly selected to be closed off whereas on the second trial, of 2 minutes duration, both arms were open. These trials were separated by a 2-minute inter-trial interval, during which time the mouse was returned to its home cage and the maze was cleaned with 70% ethanol. The time spent in each of the arms was quantified. Discrimination of spatial novelty was assessed by a preference index [21] determined as: (time in the new-time in the old arm)/(time in the new + time in the old arm).

#### Data analysis

All results are presented as mean ± *SEM* and were analyzed with SPSS 15 software (Genius Systems, Petah Tikva, Israel). Analysis of variance (ANOVA) was used to compare mnemonic treatment effects between groups. Post hoc analyses used Fisher's LSD test. A *p*-value of 0.05 or less was defined as significant statistically.

## Results

### Experiment 1: Fluid Percussion Injury in rats

#### Injury

The average injury level after FPI was 1.85 atm (range, 1.82–1.95 atm) and suppressed the return of the righting reflex an average of 449 seconds as compared with 60 seconds or less in the sham animals. Separate *t*-tests were used to compare the injury severity in atm (*t* = 1.018, *df* = 15, *p*>0.05) and righting times (*t* = 0.781, *df* = 15, *p*>0.05) of the two injured groups and revealed no significant difference in either outcome measure, indicating comparable levels of injury severity.

#### Morris water maze

**Figure 1:**
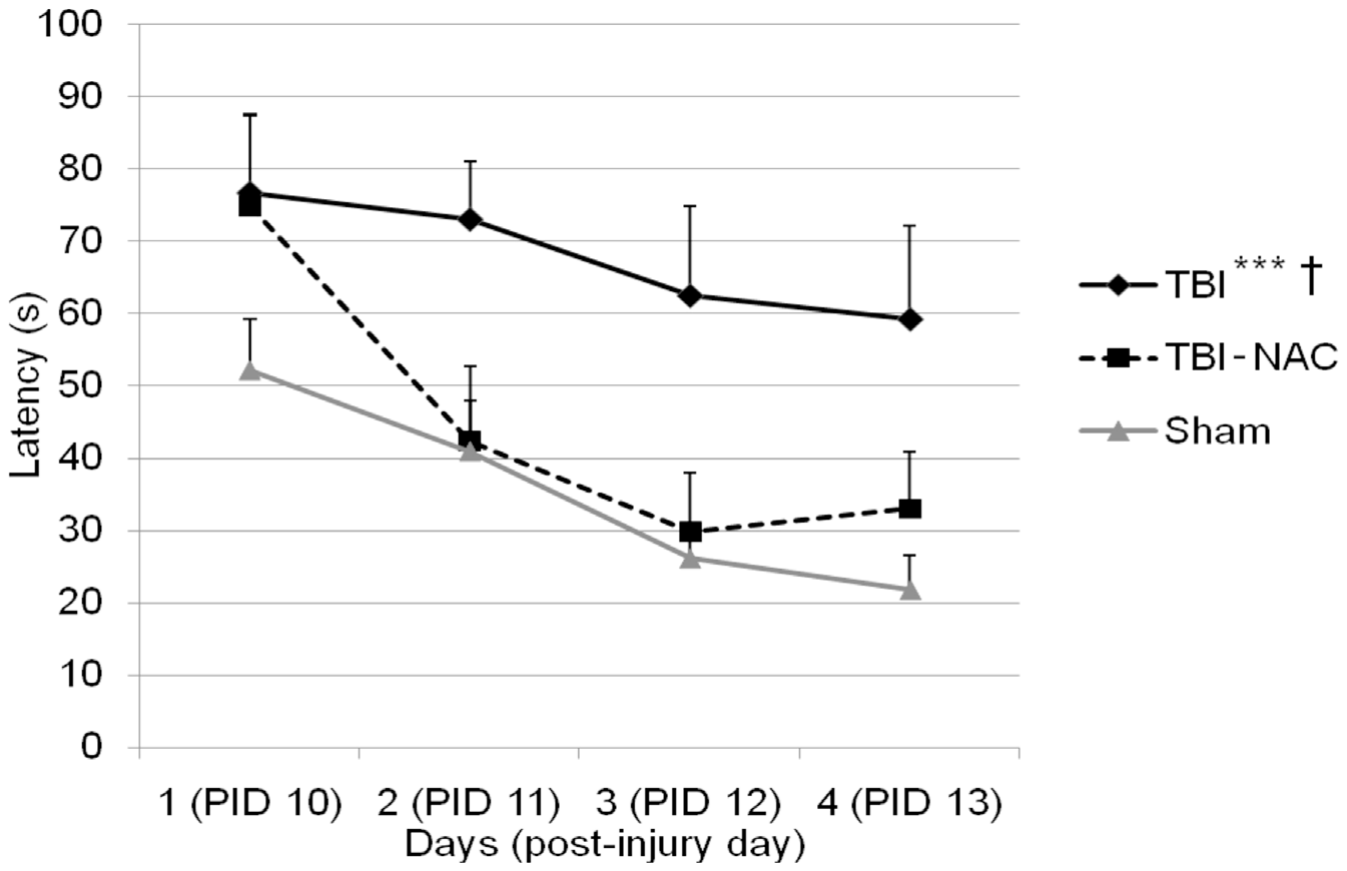
MWM performance. Post injury administration of NAC significantly improves MWM performance. MWM performance as measured by latency to reach the goal platform was compared between groups: TBI (*n* = 9), TBI-NAC (*n* = 8), and Sham (*n* = 9). Both Sham and TBI-NAC groups have significantly shorter latencies to reach the goal platform as compared to the TBI group. Additionally, treatment with NAC after TBI improved performance in the MWM that reached sham levels. Data are presented as the mean ± *SEM*. *p<.05, ***p≤.001, sham relative to TBI. † *p*<.05 TBI-NAC relative to TBI.

The latency to reach the goal platform was compared across groups during hidden platform testing in the MWM (Figure 1). A repeated measures ANOVA showed a significant difference between treatment groups, *F*(2, 23) = 7.529, *p*<0.01. Post hoc analysis using Fisher's LSD test revealed that sham animals performed significantly better during the MWM task (i.e., shorter latency to reach the goal platform) as compared to TBI, *p* = 0.001. Post injury treatment with NAC significantly improved maze performance relative to TBI, *p*<0.05. Performance in the TBI-NAC group was statistically similar to sham (*p*>0.05), suggesting that early administration of NAC ameliorates these TBI-induced cognitive deficits as assessed by the MWM task, *p*>0.05. A One-way ANOVA was used to compare overall average swim speed during hidden platform testing and revealed no significant differences between groups, *F*(2,23) = 1.016, *p*>0.05. These data indicate that motor deficits did not contribute to the observed group differences in latency to reach the platform.

**Figure 2:**
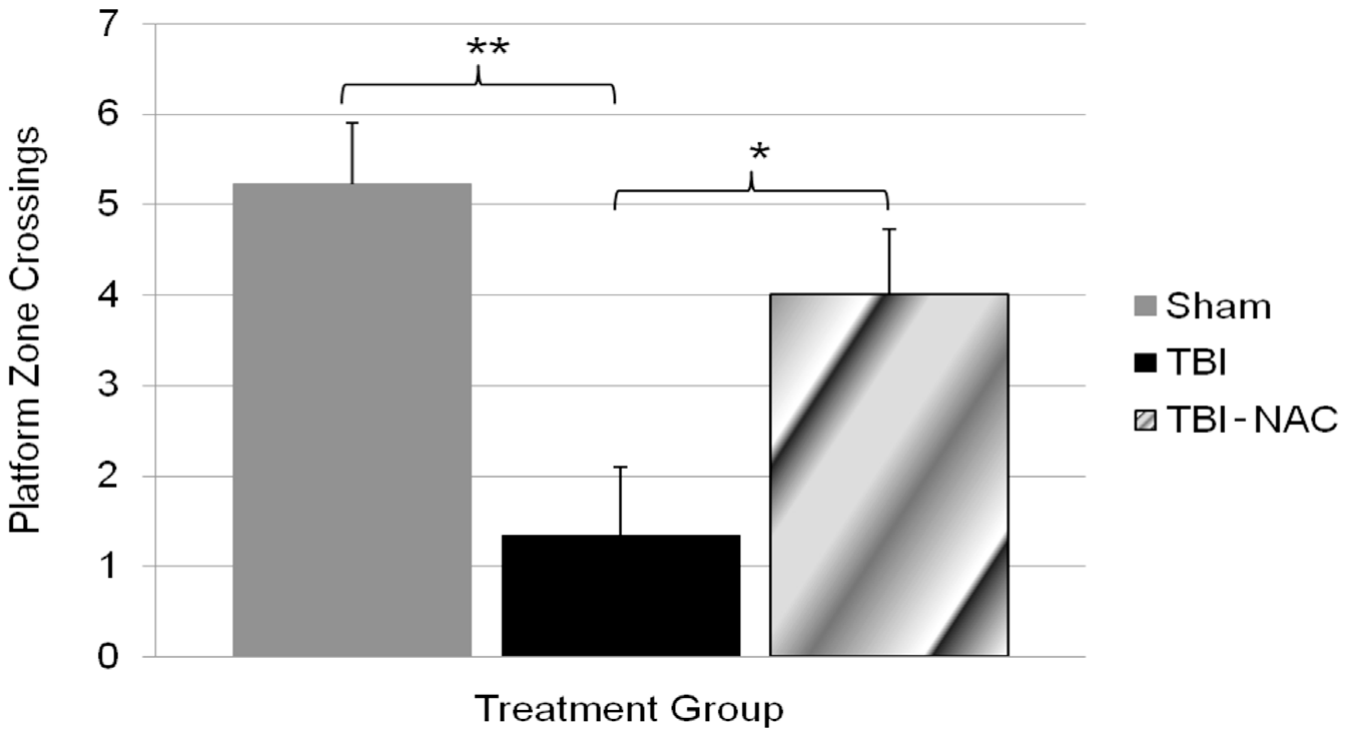
MWM platform crossing. Number of times animals crossed within a 7.5-way ANOVA showed significant differences between groups. Fisher's LSD post hoc showed that sham and TBI-NAC had significantly better retention of the platform location as compared to TBI alone. Data are presented as the mean ± *SEM*. Brackets indicate comparisons between groups. *p<0.05, **p<0.01.

The probe trial (PID 14) consisted of a single 30 second trial, with the platform removed, after the final day of hidden platform testing. The object of this test is to assess the overall learning of the platform location. Results from the probe trial are shown in Figure 2. The number of times each rat swam across the platform zone, defined as an additional 7.5 cm radius around the platform, was compared across groups. A one-way ANOVA found a significant difference in the average number of platform crossings between groups, *F*(2,23) = 7.729, *p*<0.01. Fisher's LSD analysis showed that sham and injured rats treated with NAC had significantly more platform crossings as compared to injured untreated rats, *p*≤0.001 and 0.05 respectively. There was no significant difference observed between sham and TBI-NAC groups. The visible platform test (PID 14) was performed to assess visual acuity, and motor ability to determine if performance impairment was due to a deficit in visual acuity or motor ability. A one-way ANOVA found no significant difference in the mean latencies to reach the platform between groups.

### Experiment 2: Weight Drop in Mice

**Figure 3:**
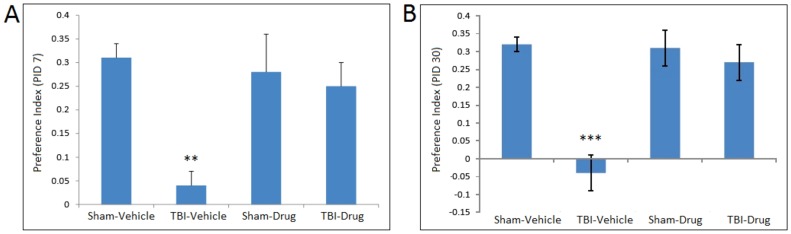
Novel Object Recognition. Preference index for the novel object in the Novel Object Recognition task across post-injury time points. Separate ANOVAs were used to compare the preference index for the novel object during the recall phase of the task. A) Weight drop injury resulted in significant object memory impairment on post-injury day (PID) 7 (A) and PID 30 (B) in injured vehicle-treated mice as compared to all other treatment groups. At both post-injury time points, post-TBI treatment with N-Acetylcysteine + topiramate (Drug) was protective against injury-induced deficits in recognition memory. Animals in the TBI-Drug group performed similarly to Sham-Vehicle and Sham-Drug groups. Values represent the mean ± *SEM*. ** *p*<0.01, *** *p*<0.001 compared to sham vehicle.

**Figure 4:**
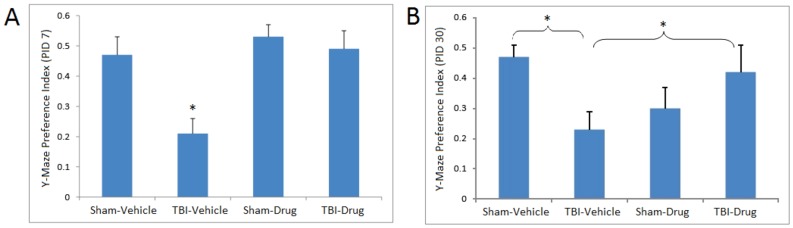
Y maze preference index. Preference index for the Y maze spatial memory task on post-injury day (PID) 7 and 30. Separate ANOVAs were used to compare the preference index for the novel arm of the Y maze during the recall trial (i.e., 2^nd^ trial). (A) Weight drop injury resulted in significant object memory impairment in TBI-Vehicle animals relative to the other three treatment groups on post-injury day (PID) 7. (B) On PID 30, mice in the TBI-Vehicle group performed significantly worse as compared to the Sham-Vehicle and TBI-Drug groups (*p*<0.05). The Sham-Drug group did not differ significantly from any of the other groups. N-Acetylcysteine + topiramate (Drug) was protective against injury-induced deficits in performance in spatial memory-dependent tasks. Animals in the TBI-Drug group performed similarly to Sham-Vehicle. Values represent the mean ± *SEM*. * *p*<0.05 compared to sham vehicle.

Novel object recognition performance showed significant drug treatment (F(1,26) = 4.50, p<0.05), TBI (F(1, 26) = 12.12, p<0.01), and treatment X TBI(F(1,26) = 10.34, p<0.01) effects in two-way repeated measures ANOVA (repeated measure: days post-TBI). Two-way repeated measures ANOVA of Y maze performance data showed significant effects of TBI (F(1, 25) = 4.37, p<0.05) and a TBI X drug treatment interaction (F(1, 25) = 9.12, p<0.01). Seven days after the injury (Figs. 3A, 4A), two way ANOVA revealed main effects of TBI (F(1, 31) = 5.94, p<0.05), and a treatment X TBI (F(1,26) = 10.34, p<0.01) interaction for novel object recognition and a TBI effect (F(1,28) = 4.11, p = 0.05) for Y maze performance. Post-hoc multiple range LSD tests demonstrated that mTBI mice exhibited lower performance than the other groups in both the novel object recognition (LSD tests p<0.01 versus the other three groups] and the Y maze (LSD tests, p<0.05 versus the other three groups) tasks. In contrast, the animals that were treated with topiramate and NAC did not differ significantly from the two control (vehicle and drug treated) groups (LSD tests). The cognitive performance impairments persisted 30 days after the trauma (Figs. 3B, 4B); two way ANOVA revealed main effects of TBI (F(1, 26) = 12.41, p<0.01), treatment TBI (F(1,26) = 7.04, p<0.05) and a treatment X TBI (F(1,26) = 7.86, p<0.01) interaction for novel object recognition and a TBI effect (F(1,28) = 7.46, p = 0.011) for Y maze performance. Post-hoc LSD tests showed that the TBI group showed significant decrements in both novel object recognition and Y maze performance, and the 30 day performance did not differ significantly from performance at 7 days. For the novel object recognition task, the TBI group showed poorer performance than each of the other three groups (LSD tests, p<0.001). For Y maze performance, the TBI group showed poorer performance than either the control-vehicle treated (LSD test, p<0.05) or the TBI-drug treated (LSD test, p<0.05) groups. The drug treated control group was intermediate and did not differ significantly from any of the other groups.

## Discussion

In this study, using two different injury models in two different rodent species, we found that early post-injury treatment with NAC reversed the behavioral deficits associated with TBI. These data suggest generalization of a protocol similar to our recent clinical trial with NAC in blast-induced mTBI in a battlefield setting [1], to mild concussion from blunt impact trauma. The use of different models in the two rodent species are predicated on both conceptual and technical reasons. Conceptually, the weight drop and FPI models span the range of mild-moderate TBI. Moreover, mice swim much more poorly than rats, and the weights needed to injure the rat brain are an order of magnitude greater than for mice. These larger weights frequently elicit skull fractures.

**Figure 5:**
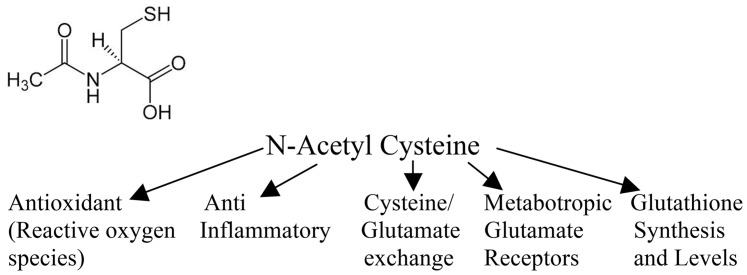
Proposed mechanism of action of N-Acetylcysteine.

These data augment a growing clinical and basic research literature on the efficacy of NAC in early treatment following mild TBI. The present study was designed to parallel a protocol used with blast mTBI in a combat setting, which included early symptomatic treatment (e.g., topiramate for headache) and NAC. The outcomes with both the rat fluid percussion model and the mouse weight drop model are consistent with the neuroprotective efficacy observed by others following a single dose of NAC in ameliorating biochemical and histological endpoints in a rat weight drop model [11] and of multiple doses in ameliorating inflammatory sequelae in an open skull dural impact rat model [9]. The antioxidant and anti-inflammatory effects of NAC [22], [23], [24], [25], [26], [27] are likely downstream consequences of inhibition of NAC-induced nuclear factor-κB-activated pathways that include cytokine cascades and phospholipid metabolism [28], which may also underlie broader efficacy of NAC in rodent ischemia-reperfusion cerebral stroke models [29], [22], [23,], a rodent sensory nerve axotomy model [30], and prevention of mitochondrial damage with loss of dendritic spines in hippocampal neurons [27]. Thus, NAC likely works on a number of levels - and clearly has antioxidant activity itself. However, it also acts as a precursor for glutathione (GSH); which is a tripeptide derived by linking the amine group of cysteine to a glycine and to the carboxyl group of the glutamate side-chain. GSH is an important intracellular antioxidant, that prevents damage caused by reactive oxygen species. GSH is synthesized within its target cells from the amino acids, L-cysteine, L-glutamic acid and glycine. Importantly, it is the sulfhydryl (thiol) group (SH) of cysteine that serves as a proton donor and is thus responsible for the antioxidant activity of glutathione. It is cysteine that is the rate-limiting factor in cellular GSH synthesis, as this amino acid is relatively rare in foods. The cellular bases for memory and regulation of motivation associated with the nucleus accumbens may also be improved via NAC-induced neuronal activation of cysteine-glutamate exchange, augmented by indirect effects of NAC on metabolic glutamate receptors, mGluR2/3 and mGluR5, as reported for amelioration of cocaine-induced disruption of memory and regulation of motivation in rodents [31]. These multiple mechanisms of NAC actions are diagrammed in Figure 5.

The therapeutic bioavailability of systemic N-acetylcysteine following TBI is a function of both the regulation of free levels in the blood and permeability of the blood-brain barrier. Because free cysteine is regulated tightly by the mammalian liver, via mechanisms that reach a new steady-state within 24 hours [32], repeated bolus doses are expected to be most effective for affecting circulating free NAC levels. However, the relative impermeability of the normal blood-brain barrier to NAC [24] implies that local CNS bioavailability would be a natural consequence of intracranial vascular disruption in mTBI, either acutely during vascular remodeling after injury [33] or a delayed leakiness of the blood-brain barrier from neuroinflammatory processes [33], [34]. Moreover, supporting the potential role of GSH in the effects of NAC, it has been shown that, despite its poor penetration into the CNS, NAC can significantly elevate GSH levels in brain after oxidative stress [35], [36] and GSH deficiency [37]. Moreover, it has recently been shown that, in a unique animal model of mTBI using thinning of the skull and compression, that glutathione from the periphery can enter the brain and exert neuroprotective activity [38].

The importance of vascular damage in mTBI has been recently emphasized by Franzblau et al [39] as a mechanistic link between traumatic brain injury and the subsequent development of Alzheimer's Disease. Upregulation of the “Alzheimer's Disease gene set” after the weight drop model in mice has been recently reported by Tweedie et al [40]. In addition, recent studies by Acosta et al [41] suggest that neuroinflammation associated with traumatic brain injury may suppress hippocampal neurogenesis, with in turn, may underlie some of the cognitive deficits seen in this disorder. The improved clinical outcomes after early NAC treatment for blast TBI [1] are consistent with the hypothesis that vascular effects of TBI facilitate selective delivery of NAC to affected sites.

In summary, this paper documents the efficacy of NAC in reversing or preventing cognitive abnormalities in rodent models of mild to moderate TBI. Future preclinical studies are needed to further define the mechanism of action, leading to more effective therapies in man. We also can now begin to consider clinical work in a human model since the current set of experiments attempted to approximate considerations needed in a clinical study by utilizing and accepted standard of care in the animals in experiment two.
